# Progression of health‐related quality of life of patients waiting for total knee arthroplasty

**DOI:** 10.1111/jep.13388

**Published:** 2020-03-22

**Authors:** Ki Wai Ho, Gerald Pong, Wai Chin Poon, Kwong Yin Chung, Yan‐Yan Kwok, Kwok Hing Chiu

**Affiliations:** ^1^ Department of Orthopaedics and Traumatology The Chinese University of Hong Kong Hong Kong; ^2^ Department of Orthopaedics and Traumatology Prince of Wales Hospital Shatin Hong Kong; ^3^ School of Nursing, Li Ka Shing Faculty of Medicine The University of Hong Kong Hong Kong

**Keywords:** end‐stage osteoarthritis, HRQoL, quality of life, SF‐36, total knee arthroplasty, WOMAC

## Abstract

**Background:**

Total knee arthroplasty (TKA) remains the surgical gold standard treatment for patients suffering from end‐stage osteoarthritis (OA) of the knee. However, due to the high demand and scarce medical resources, the waiting time for surgery is astoundingly lengthy. Controversies are shown in numerous studies, on whether physical functionality and mental status decline or remain stable over the waiting period. This study aims to evaluate the progression in patients suffering from end‐stage OA while on the waiting list for TKA.

**Methods:**

One hundred and twenty‐seven patients suffering from end‐stage OA who were on the TKA waiting list were prospectively recruited from our orthopaedics specialist clinic. They were assessed once a year for 2 years or until surgery. The Western Ontario and McMaster University Osteoarthritis Index (WOMAC), SF‐36 self‐rated questionnaire and 15D health‐related quality of life (HRQoL) questionnaire were used as outcome measurements for functionality and disability assessment.

**Results:**

Patients on the waiting list for TKA showed a progressive increase in pain and disability level within the first year (*P* = .035). Those patients waiting for more than 2 years showed worsening HRQoL (*P* < .05) as time progressed. However, no significant difference was observed between the first and second years.

**Conclusions:**

A decline in functionality and increase in disability were shown in follow‐up assessments conducted every year. However, a plateau effect is observed with end‐stage disease. This emphasizes that more active conservative management programmes should be introduced and implemented while patients are enlisted on the TKA waiting list. Moreover, timely surgical intervention can improve patients' overall function.

**Trial registration:**

This study involved human participants and reports health‐related outcomes concerning the HRQoL in patients with end‐stage OA of the knee. Thus, it was registered, retrospectively, as a clinical trial under the U.S. National Library of Medicine ClinicalTrials.gov (https://clinicaltrials.gov/) on March 4, 2018.

## 1 INTRODUCTION

Osteoarthritis (OA) is one of the most common chronic degenerative joint disorders in the ageing population. It limits joint movements, induces pain and stiffness, and often leads to exacerbation in the surrounding degenerative changes to tissues, causing disability over time. The prevalence of radiological knee OA rises in proportion with age and sex. The estimated generality of OA knee joint disease is commonly reported as 64.1% and 71.4% in those aged 60 and 70 years and above, respectively, while females have a greater prevalence rate than men.^1^ OA patients often encounter swelling, stiffness and pain in their lower extremities, and they usually respond to these symptoms by decreasing physical activities, leading an overly sedentary lifestyle.^2^, ^3^ In turn, this inactivity often leads to muscle loss and weight gain. Disease progression is usually slow, but patients become more symptomatic in the end stage of the condition and consequently suffer more pain and disability.^4^

Total knee arthroplasty (TKA) is a common procedure for end‐stage knee arthritis that has shown significant improvement in terms of functionality, pain and quality of life.^5^, ^6^, ^7^, ^8^, ^9^ It remains the gold standard treatment for severe OA of the knee and has gained much public awareness in most third‐world developed countries. In places like Hong Kong, the local public health care system provides TKA procedures at a very low cost, but with limited resources, it is often difficult to keep up with the demand from an ageing population.^10^ Delaying surgery would further deteriorate bodily functions. Years of postponement deprive patients of the full beneficial potential of TKA, reducing the mobility function in rehabilitation.^11^ The present study facilitates the demonstration of evidence‐based management with timely knee replacement intervention practices at the level of consensual decision‐making by the orthopaedic surgeon.

Patient‐reported outcome measures (PROMs) have been used widely in studies to assess outcomes in osteoarthritic patients,^12^, ^13^, ^14^ and have shown an exceptional improvement in communication and better decision‐making between doctors and patients in addition to improving patient satisfaction with care.^15^, ^16^, ^17^, ^18^, ^19^ Most if not all of these measures have displayed favourable amounts of validity and reliability, which are necessary for appraising patient knee functionality and health‐related quality of life (HRQoL).

Any difference in HRQoL or self‐perceived knee functionality is potentially crucial for conducting a comprehensive assessment of OA in both a clinical and research setting; understanding the difference would enable health care providers to have a more comprehensive understanding of the impacts of chronic disease from the patient's perspective and, most importantly, deliver a more holistic health care service.

The aims of this study were to investigate whether there are any differences in symptomatic knee OA patients in regard to their HRQoL, self‐perceived knee functionality and their mental status while waiting for a TKA procedure over an extended period.

## 2 MATERIALS AND METHODS

All procedures performed in studies involving human participants were performed in accordance with the ethical standards of The Joint Chinese University of Hong Kong—New Territories East Cluster Clinical Research Ethics Committee, and were approved by the committee on November 18, 2013 under reference number 2013.381. This study has been performed in compliance with the Declaration of Helsinki and its later amendments or comparable ethical standards. As it involves human participants, informed consent was therefore obtained from all individuals who participated in this study.

This was a prospective longitudinal study, involving patients with symptomatic OA treated at the Prince of Wales Hospital, Hong Kong from March 2013 to December 2016. By universal sampling, patients visiting the Li Ka Shing Orthopaedic Specialist outpatient clinic who had experienced symptomatic OA were screened by a research nurse or a research assistant based on the inclusion criteria. Inclusion criteria were (a) diagnosed with symptomatic OA of Kellgren‐Lawrence classification Grade III to IV, (b) enlisted on the TKA surgical waiting list, (c) ethnic Chinese and (d) fluent in Cantonese. The exclusion criteria were (a) evidence of cognitive dysfunction like dementia, (b) surgery scheduled within 30 days and (c) severe comorbidity precluding participation.

Over 300 patients with OA symptoms of the knee were screened for study eligibility; a total of 127 patients were recruited at baseline. Consecutive clinic consultations facilitated the collection of follow‐up data; when clinical attendance was not observed, HRQoL and behaviour status of research subjects were determined by a telephone evaluation survey. A few patients refused to spend their time on the telephone survey; those were treated as incomplete or lost to follow‐up.

## 3 OUTCOME MEASURES

The outcome measurements for this study are represented by the symptoms, functionality scores and self‐rated HRQoL represented by two separate questionnaires. The 36‐Item Short Form Survey version 2 was used to measure HRQoL, while the Western Ontario and McMaster Universities Osteoarthritis Index was administered to measure patients' pain and functionality. Assessments were conducted on the first day when recruited to the study. Subsequent assessments were carried out at 1 year and 2 years after the first assessment. Participants that were unable to attend their yearly consultation were then contacted via telephone by research staff to carry out the assessment and inquire about their current HRQoL and behavioural status.

### 3.1 36‐Item Short Form Survey version 2

The 36‐Item Short Form Survey version 2 (SF‐36v2) is an instrument designed to measure health concepts relevant across age, disease and treatment groups. It is a validated instrument that has been administrated often to measure the HRQoL and disability in different cohorts.^20^ The questionnaire uses 36 separate questions that involve the patient's physical difficulty, functionality, and social and mental function. These areas are summarized into eight scaled score domains (physical function, role physical, bodily pain, general health, vitality, social function, role emotional and mental health), which are the sums of the questions in their respective sections. Each domain is directly transformed into a 0 to 100 scale with the assumption that each of the 36 questions carries equal weight. The scoring is summarized, with the lower the score the greater the disability and vice versa.

### 3.2 Western Ontario and McMaster Universities Osteoarthrosis Index

Western Ontario and McMaster University Osteoarthritis Index (WOMAC) is a common standardized questionnaire that assesses the status of patients with OA of the knee and hip. The questionnaire comprises three domains with 24 questions in total, with 5 questions on pain, 2 questions on stiffness and 17 questions on physical function. Each question is scaled from 0 to 4, with 0 being “none” and 4 being “extreme”. These scores are then summed up into their respective domains and added together to produce a total score scaled between 0 and 96. The scoring is summarized, with the higher the score the more pain, stiffness and physical disability.

## 4 STATISTICAL ANALYSIS

The data collected were analysed using IBM SPSS Statistics 20. Normality of the data was assessed manually. Age, body mass index and duration of symptoms were entered as continuous variables; others were entered as binary categorical variables. Descriptive statistics including mean and standard deviations were calculated and reported for all SF‐36 and WOMAC sub‐domains collected from patients' follow‐up. An ANOVA with repeated measures was used to compare the differences between each yearly follow‐up.

## 5 RESULTS

A total of 127 patients were recruited initially. However, as it was not possible to contact a few patients, the dataset of fully completed records was statistically processed for 86 patient participants. The cohort consisted of 61 females (70.9%) and 25 (29.1%) males, with a mean age of 67.4 (SD 11.4) years and BMI of 27.1 kg/m^2^ (INQ 23.6‐29.5). Average waiting time for patients was 2.5 years. The majority of those recruited addressed the knee joint (89.5%) as the index joint and all patients had Kellgren and Lawrence scores of Grade III to IV based on the inclusion criteria for this study. In general, patients showed a gradual increase in disability and pain over the course of the 2‐year study period. Furthermore, patients also showed a worsening HRQoL, based on their responses to the SF‐36v2 questionnaire.

### 5.1 36‐Item Short Form Survey version 2

#### 5.1.1 Physical component domain scores

Patients showed no significant difference in their first‐year physical component score (Table 1). However, they showed significant deterioration in their general physical component score after or during their 2‐year follow‐up (PCS, −7.95, *P* = .00). Individual domains showed a significant deterioration in physical function (PF) in both first‐ (−2.54, *P* = .02) and second‐year (−11.40, *P* = .00) follow‐ups. A significant deterioration in role physical (RP) and increase in bodily pain (BP) were also seen, but only in second‐year follow‐ups (RP, −22.19, *P* = .00) (BP, −8.08, *P* = .00). No significance was measured in the general health domain at either follow‐up.

**TABLE 1:** Health‐related quality of life questionnaire, Short Form 36 version 2

SF‐36v2	Initial	Post 1 year and initial			Post 2 years and initial		
Domain	Average	Difference	SE	*P*‐value	Difference	SE	*P*‐value
Physical function	30.638	−2.542	0.913	**.020**	−11.394	1.787	**.000**
Role physical	33.816	1.134	1.655	1.000	−22.188	1.706	**.000**
Bodily pain	34.793	−0.853	1.177	1.000	−8.078	1.437	**.000**
General health	37.306	−1.130	1.157	.994	1.124	1.431	1.000
Vitality	42.295	0.617	1.401	1.000	0.147	1.630	1.000
Social function	44.684	−2.133	1.958	.838	−3.550	2.247	.354
Role emotional	33.502	−0.142	1.854	1.000	−19.257	1.843	**.000**
Mental health	46.722	−5.363	1.607	**.004**	3.743	2.630	.475
Physical component score	31.960	0.337	0.885	1.000	−7.954	1.073	**.000**
Mental component score	44.288	−1.733	1.354	.612	−7.215	1.275	**.000**

#### 5.1.2 Mental component domain scores

Patients showed no significant deterioration in their first‐year mental component score. However, they showed a significant deterioration in their general mental component score at 2‐year follow‐up (MCS, −7.22, *P* = .00). Individual domains showed a significant deterioration in role emotional at second‐year follow‐ups (RE, −19.26, *P* = .00). Patients showed a significant deterioration in mental health only at their first‐year follow‐up (MH, −5.36, *P* = .00); however, an insignificant improvement was shown at their second‐year follow‐up. No statistically significant values were obtained for either the vitality or social function domain.

### 5.2 Western Ontario and McMaster University Osteoarthritis Index

Patients showed a statistically significant difference in total and percentage score in their first‐ (total, +8.44, *P* = .00; percentage, +8.79, *P* = .00) and second‐year (total, +11.59, *P* = .00; percentage, +12.08, *P* = .00) follow‐ups (Table 2). They also showed a significant worsening in both pain and functionality scores. In regard to the pain score domain, patients showed a significant average increase of 1.24 in their first year (*P* = .00) and a higher pain score, with a significant increase of 2.08 compared to the initial value (*P* = .00). For physical functionality, patients showed an increase in disability at their first‐year follow‐up, with a 6.80‐point increase (*P* = .00) and even greater disability in their second year, with a significant increase in disability score of 9.16 compared to their initial score (*P* = .00). In addition, no significant difference was measured within stiffness score domains at either follow‐up.

**TABLE 2:** Pain, stiffness and physical functionality questionnaire, Western Ontario and McMaster Universities Osteoarthritis Index (WOMAC)

WOMAC	Initial	Post 1 year and Initial			Post 2 years and Initial		
Domain	Average	Difference	SE	*P*‐value	Difference	SE	*P*‐value
Total score	50.85	**8.442**	1.341	**.000**	**11.593**	1.341	**.000**
Pain score	11.57	**1.244**	0.333	**.001**	**2.081**	0.327	**.000**
Stiffness score	4.02	0.395	0.188	.116	0.360	196.000	.207
Function score	35.26	**6.802**	1.007	**.000**	**9.163**	1.042	**.000**
Percentage	52.97	**8.794**	1.397	**.000**	**12.076**	1.397	**.000**

## 6 DISCUSSION

The present study investigates the health status and HRQoL of patients with OA while waiting on the surgical waiting list for TKA. During the prolonged period of queuing, they often undergo a tremendous amount of physical and mental impairment which should be addressed, as it is crucial for disease complications to be cured. We believe this is the first longitudinal study to document the progression of the health condition and HRQoL of patients with end‐stage OA waiting for TKA with a waiting period of more than 2 years.

Our results have demonstrated that patients with degenerative joint illness of the knee experience a significant increase in disability, decline in functionality, mental impairment and a worsening of HRQoL while they are waiting for their scheduled TKA surgery. Based on the SF‐36v2, patients show significant deterioration in the physical function and mental health domains. Although every domain has equal weight in the physical and mental components within the questionnaire, the significance of these two domains is not to be dismissed lightly. Specifically, mental health deterioration and impairment have been investigated as one of the major contributions of the increase in disability in OA. Patients who experience severe OA symptoms such as pain and stiffness experience depression, causing a more sedentary lifestyle leading to more disability, creating a vicious cycle.^21^, ^22^, ^23^, ^24^ WOMAC shows a significant increase in their total, percentage, pain and physical functionality score during their first‐year follow‐up, with physical function score being highest in the three domains that were measured. During their second‐year follow‐up, patients' HRQoL further deteriorated, adding role physical, bodily pain and role emotional as significant domains, clearly showing that patients that wait longer for the surgical procedure will have more disability, mental impairment and pain and a greater deterioration in general well‐being. WOMAC validates this claim by showing an even bigger difference from their initial values, with the total score being an astounding 12% increase from the baseline value.

These findings are consistent with several previous studies which have reported a worsening in patient status after being on the surgical waiting list.^24^, ^25^, ^26^, ^27^, ^28^ Ackerman and colleagues had similar investigation methods, using a quality of life questionnaire, WOMAC and a psychological distress scale to show any potential decline in health and social well‐being. The results collected from their study showed that over 60% of the 134 patients showed a decline in their health status and conditions.^25^ However, the limitation of the study is that most patients that were included only had an average waiting time of less than a year, so the true long‐term effects of waiting more than 12 months are not reflected. In contrast, our study subjects, when recruited, had been waiting at least 1 to 2 months and at most almost 5 years, putting our study at an advantage as the results obtained can reveal a more constructive layout of disease progression of patients in waiting after 1 year. Some studies have shown conflicting findings when comparing their HRQoL. In particular, Hirvonen et al recruited 133 patients to assess their HRQoL while waiting for scheduled arthroplasty, and showed no difference or worsening in patients' quality of life while waiting.^29^ However, the study utilized a different questionnaire/instrument to assess patients' HRQoL, which may or may not be comparable with our current findings. The questionnaire used by Hirvonen et al had 15 questions, compared to the 36‐question questionnaire used in this present study for HRQoL, which may show a more accurate, conclusive and all‐round assessment of patients' quality of life.

In a prospective cohort study done by Fortin and colleagues in the United States, clinical outcomes, self‐reported health status and HRQoL of 222 OA patients who had undergone TKA were investigated. This study showed that subjects with the worst function and pain at the time of surgery had a comparatively worse functional outcome 2 years after surgery, which suggests that the timing of surgery may be associated with a better outcome.^30^ Comparing the long waiting time for TKA in Hong Kong, this study has provided evidence‐based information for the health care authorities to refine the current structure and tackle the major flaws within the health care system. Based on our findings, all patients should ideally receive TKA within the first year of waiting, as there was a statistically significant deterioration in pain and functional impairment during their first year of waiting. It is strongly advised that patients should not exceed 2 years of waiting, to avoid any further deterioration of their pre‐operative health status and to maximize their potential post‐operative functional and mental outcomes, and recover to their fullest extent. However, this is neither realistic nor feasible in countries that are overpopulated with elderly patients. We may also see a ceiling effect with patients who have been waiting for more than 2 years on the waiting list, as the current outcome assessment is neither accurate nor sensitive in detecting differences in patients with extremely low scores.

Non‐invasive management for OA, specifically exercise therapy, has been widely investigated. However, most of these studies aim to rehabilitate patients who have undergone TKA procedures for a faster recovery post‐operatively. Investigations of the effects of non‐invasive intervention on patients who have not undergone surgery are still limited. Given that OA is an illness with high comorbidities (ie, cardiac diseases, hypertension, obesity, etc), these studies that have incorporated exercise therapy as an intervention for OA have also described the restrictions and contraindications that OA patients face when performing these exercises. A prime example is that restrictions and contraindications such as pain and fatigue due to high‐activity exercises may discourage obese patients, causing a vicious cycle and defeating the original purpose of being more active. Meditative exercises such as Tai Chi have grown in popularity among the older adult population due to their simplicity. Tai Chi is a complex multi‐component mind‐body exercise that combines deep diaphragmatic breathing with slow, gentle and graceful movement. The benefits of Tai Chi have been well documented on bone health, fall prevention and even symptoms that are related to OA such as a decline in pain and stiffness and increase in physical functionality.^31^, ^32^, ^33^, ^34^, ^35^, ^36^ However, the utilization of such exercises is still absent in common pre‐habilitation and rehabilitation programmes. Given the trendy and highly beneficial effects of Tai Chi exercises in OA patients, we suggest that Tai Chi exercises should be included in OA exercise therapy regimes to slow down the progression of OA symptoms while patients are on the waiting list for TKA procedures. In addition, these exercise therapies can also act as a pre‐habilitation programme so as to exhibit a better post‐operative outcome.^37^

Several limitations have to be taken into account while interpreting the results of the current study. Although this longitudinal study was the first to have 2 years of follow‐up data on patients, it was a victim of selection bias as subjects were only recruited from a single tertiary referral centre; however, this is not an uncommon scenario, as there are long waiting times in other centres in our region. Referral time from general clinics to the orthopaedic specialist clinics was not taken into account due to difficulties in terms of recruiting subjects at that time. Patients might have been on the waiting list for consultation for a long period of time before being put on the surgical waiting list, and symptom severity and disease progression might vary during the time of waiting. In addition, the questionnaires measuring the patients' HRQoL are mostly self‐reported. Although both WOMAC and SF‐36 are legitimate and validated measuring tools for detecting sensitivity to changes,^38^, ^39^, ^40^ performance or physical measurements should be executed to have a more reliable outcome.

## 7 CONCLUSION

This study has generated valuable clinical implications and given evidence‐based information for the medical field and policy makers to reconstruct the current health care system. Based on the data obtained, it is suggested that the waiting time for joint replacement surgery should not exceed 2 years to prevent a further deterioration of pre‐operative status among patients with end‐stage knee OA and maximize their potential post‐operative functional outcomes.

## CONFLICT OF INTEREST

The authors declare no competing interests.

## AUTHOR CONTRIBUTIONS

Author Ki W. Ho performed the clinical examination of functionality and disability assessments on patients and was a duty as a major contributor at methods planning for this study. Gerald Pong performed interpretation of patient data analysis based upon the WOMAC, SF36 and HRQoL index, and was duty a major contributor in writing of the manuscript. Wai‐Chin Poon provided additional sources of literature findings in responsible for further drafting of the revised paper version, and together with the preparation of journal publication works involved. Kwong Y. Chung, Yan‐Yan Kwok and Kwok H. Chiu contributed their mutual recognition of efforts at subject recruitment, clinical data collection, patient care enforcement during the entire process of this study. All authors provided critical feedback and helped shape the research, analysis and manuscript. All authors have read and approved on this finalized manuscript version.
